# Extraction, Characterization, and Antimicrobial Ability of Chitosan and Edible Oil From Ugandan Winged Termites

**DOI:** 10.1002/fsn3.71013

**Published:** 2025-09-26

**Authors:** Babirye Khadijah, Ammar Ahmad Khan, Shahrul Razid Sarbini, Aqsa Abid

**Affiliations:** ^1^ University Institute of Food Science and Technology, Faculty of Allied Health Sciences The University of Lahore Lahore Pakistan; ^2^ Department of Food Science and Nutrition, Faculty of Agribusiness and Natural Resource Sciences Islamic University in Uganda Mbale Uganda; ^3^ Halal Assurance Institute Uganda Kampala Uganda; ^4^ Faculty of Agricultural Science and Forestry Universiti Putra Malaysia Kampus Bintulu Sarawak (UPMKB) Bintulu Sarawak Malaysia; ^5^ Halal Product Research Institute Universiti Putra Malaysia Serdang Selangor Malaysia; ^6^ University Institute of Physical Therapy, Faculty of Allied Health Sciences The University of Lahore Lahore Pakistan

**Keywords:** antimicrobial properties, biofunctional food, chitin extraction, edible oil, green extraction techniques, insect flour

## Abstract

Edible oil and chitosan were extracted from the flour of four edible winged termite species (*Macrotermes subhylanus*, *Macrotermes bellicosus, Pseudocanthotermes spriniger*, and *Odontotermes lateritius*), characterized for physico‐chemical properties, and the antimicrobial activity of extracted chitosan was assessed against *Staphylococcus aureus* , *Escherichia coli* , and *Candida albicans* . Mechanical pressing yielded the significantly highest oil quantity (37.42%–38.50%) throughout all the species, followed by microwave‐assisted oil extraction. Overall, the significantly lowest oil yield was observed in the conventional soxhlet extraction method (8.05%–18.92%). Generally, the extracted termite oils appeared as clear, golden‐to‐light yellow liquids. Oil solidification temperature ranged from 10.1°C to 12.9°C, whereas the refractive index ranged from 1.23 to 1.46. Mechanically pressed oil produced the highest iodine (111–121 g iodine/100 g oil) and saponification value (110.41–113.92 mg KOH/g). The total cholesterol content of termite oils ranged from 24.98 to 39.00 mg/100 mL, and ultrasound‐assisted oil extraction yielded the lowest total cholesterol content. *M. bellicosus* oil had the highest total cholesterol content. Fermentation yielded the highest demineralization percentage (93.8%–96.0%), whereas the chemical method produced the highest deproteinization percentage (42.59%–49.71%) and chitosan yield (6.05%–7.00%). *M. subhyalinus* and *M. bellicosus* produced higher chitosan yields than other termite species. Termite chitosan showed the highest antimicrobial activity against *E. coli* , followed by *S. aureus* . Chemically‐extracted chitosan exhibited higher antimicrobial activity than enzymatically extracted chitosan. From the present study, edible winged termites can be utilized as an unconventional source of chitosan for antimicrobial activity and low cholesterol edible cooking oil.

## Graphical abstract

This study extracted edible oil and chitosan using both chemical and green extraction techniques, from four species of edible winged termites. Yield and efficiency of the extraction techniques were compared, the extracted products characterized for physico‐chemical properties, and the antimicrobial ability of chitosan assessed against a gram‐positive bacteria, gram‐negative bacteria, plus yeast. From the results, edible winged termites can be utilized as an unconventional source of chitosan for antimicrobial activity and low cholesterol edible cooking oil.

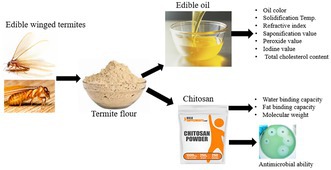

## 1 Introduction

Following the inclusion of edible insects in the EU's novel food regulations‐EU 2015/2283 (European Commission 2015), many edible insect companies have been opened in different countries to facilitate the supply of safe, innovative insect foods and ingredients to consumers worldwide (Tzompa‐Sosa et al. 2019). Several online suppliers of insects in whole form, paste/ground, flour or powders, insect oil, protein fractions, and chitin for further application as food ingredients are currently available (Juelicher 2017; Khadijah 2025). In addition to being used as a nutritious food, several industrial products, such as edible oil and chitin, can be obtained from edible termites (Babarinde et al. 2020; Khadijah et al. 2024; Kinyuru 2020). Recently, consumer demand for high‐quality edible oils with nutritional benefits has increased (Gharibzahedi and Altintas 2022). Insect oil has emerged as a prospective eco‐friendly source of edible oils. Several green technologies have emerged for insect oil extraction, including high hydrostatic pressure processing, microwave‐assisted extraction, ultrasonication, and enzyme‐assisted extraction. In addition to a higher extraction yield of insect oils, such technologies utilize comparatively little solvent and reduce the extraction duration in comparison with conventional extraction techniques (Gharibzahedi and Altintas 2022).

Existing literature on termite nutritional profiling reveals that edible winged termites are naturally rich in oil (mono‐ and polyunsaturated fatty acids), and this oil is likely to have very low cholesterol and thus is healthier than oil of animal origin (Khadijah et al. 2024). Reports indicate that termite oil might be similar to or even superior to plant oils in terms of quality (Alen et al. 2017; Ekpo and Onigbinde 2007; Kinyuru 2020, 2009). However, few studies have focused on the extraction and characterization of termite oil for edible use or industrial applications (Ajayi 2012; Kinyuru 2020, 2009). Tzompa‐Sosa et al. (2014) indicated that the extraction method affects the quantity of insect oils extracted. Additionally, there is a need to evaluate the potential of termite oil for the development of functional foods (Kinyuru 2009). From our literature search, a few countries such as China and Kenya have attempted to develop functional foods from edible termites (Chen et al. 2009; Feng et al. 2018; Siddiqui et al. 2023). Physico‐chemical characterization of insect ingredients is vital because it determines their functionality and food‐related applications (Tzompa‐Sosa et al. 2019).

Similar to other insects, termites are characterized by a chitinous exoskeleton and can thus be utilized as an unconventional source of chitosan for several applications in food technology (Khadijah 2024; Mohan et al. 2020). Rehman et al. (2023) reported that the global chitin plus chitosan market will increase rapidly and is predicted to be as high as $12.3 billion by 2027. Chitosan production is primarily done from marine sources, mainly crustaceans, but recently, edible insects are increasingly explored as promising sources (Khadijah et al. 2024; Mohan et al. 2020; Rehman et al. 2023) because they are more readily available, are easy to acquire in larger quantities, can provide comparatively similar and sometimes higher chitosan quantities, and have shorter reproductive cycles than crustaceans (Espinosa‐Solís et al. 2024; Mohan et al. 2020; Rehman et al. 2023). Importantly, it is possible to rear insects on organic side streams and waste, thus increasing economic sustainability (Rehman et al. 2023). The characterization of chitosan from insects is vital because it determines the functionality of chitosan in industrial food applications (Mohan et al. 2020).

In addition, previous reports have indicated several biological properties of chitin and chitosan, such as antitumor/anticarcinogenic and antimicrobial activity (Abdelaziz et al. 2024; Albalawi et al. 2023; El‐Fakharany et al. 2025; Khadijah et al. 2024). Previous studies have reported that chitin and its derivatives reduce the proliferation of harmful microbes such as enteropathogenic *Escherichia coli* , *Salmonella typhimurium* , *Klebsiella pneumoniae* , *Acinetobacter baumannii* , *Staphylococcus aureus* , and *Streptococcus pneumoniae* in the human stomach and lungs (Abdelaziz et al. 2024; El‐Fakharany et al. 2025; Fernandes et al. 2008). Owing to its antibacterial effect, chitosan can be used as a food active edible packaging (Hamed et al. 2016).

Chitin extraction or production conventionally involves the use of chemicals that are strong acids such as HCl and strong bases such as NaOH (Rehman et al. 2023). In addition to being expensive, these chemicals are hazardous, and their high concentrations commonly pollute the environment (Psarianos et al. 2022; Rehman et al. 2023). In light of these concerns, green extraction techniques are currently being explored, including demineralization through citric acid treatment, microwave treatment or fermentation with lactic acid bacteria, deproteinization through treatment with a protease enzyme or proteolytic bacteria, and use of the chitin deacetylase enzyme for the transformation of chitin into chitosan (Espinosa‐Solís et al. 2024; Lagat et al. 2021; Psarianos et al. 2022). Comparatively, green extraction techniques are eco‐friendly and cheaper because of the small quantity of solvents involved, require low energy input, are simpler to manipulate, and have high reproducibility (Lagat et al. 2021; Rehman et al. 2023).

To the best of our knowledge, extraction and characterization of termite chitosan has so far been attempted by Asad et al. (2024), who used a chemical extraction method but did not reveal whether winged termites were the ones used (termite samples were sourced from wood; basing on this habitat, we are convinced these were most likely soldier termites, not flying termites). No reported study exists yet on extraction and characterization of chitosan from edible winged termites, exploration of their antimicrobial ability, or a comparison between and among species. Comparison of the chitosan yield among different extraction methods is another virgin research field that merits further investigation. Moreover, most studies investigating the antimicrobial activity of insect chitosan do not separately assess the activity of acetic acid alone, the commonly used solvent; yet it is in itself antibacterial and can affect chitosan activity during the investigation. The present study, therefore, aimed at (i) extracting termite oil from flour of different edible winged termite species, comparing oil yield from different extraction techniques, and characterizing the extracted oil, (ii) extracting edible winged termite chitosan, comparing the extraction efficiency and chitosan yield from different extraction techniques, and characterizing the extracted chitosan, and (iii) assessing the in vitro antimicrobial ability of extracted chitosan against gram‐positive bacteria, gram‐negative bacteria, and yeast.

## 2 Materials and Methods

Representative samples of sun‐dried marketed ready‐to‐eat edible winged termites were purchased from local outdoor markets in Uganda (Owino, Tororo, Mbale, Kibuye, and Lira markets), located in different regions (Eastern, Northern, and Central regions) during the swarming period of the termites (September 2024). The markets from each of the selected regions and the market vendors were both selected by simple random sampling. Species identification revealed a total of four species of edible winged termites (*Macrotermes subhylanus*, *Macrotermes bellicosus, Pseudocanthotermes spriniger*, and *Odontotermes lateritius*), which were converted into flours. Flours were independently subjected to oil and chitosan extraction. Details of the termite species' identification, conversion of termites into flour, proximate composition, and nutritional profiling for each termite flour species are given in our previous work (Khadijah et al. 2025); proximate analysis of termite flours from the species under study showed a crude protein content of 50.74%–57.96%, carbohydrate content of 20.14%–28.79%, crude fat content of 8.05%–18.92%, moisture content of 5.13%–6.82%, total ash content of 6.44%–7.94%, crude fiber content of 5.28%–5.82%, and an energy value of 113.67–157.37 kcal/100 g.

### 2.1 Edible Oil Extraction and Characterization

#### 2.1.1 Oil Extraction

Termite oil extraction was carried out by four methods: conventional and green extraction techniques.

**FIGURE 1:**
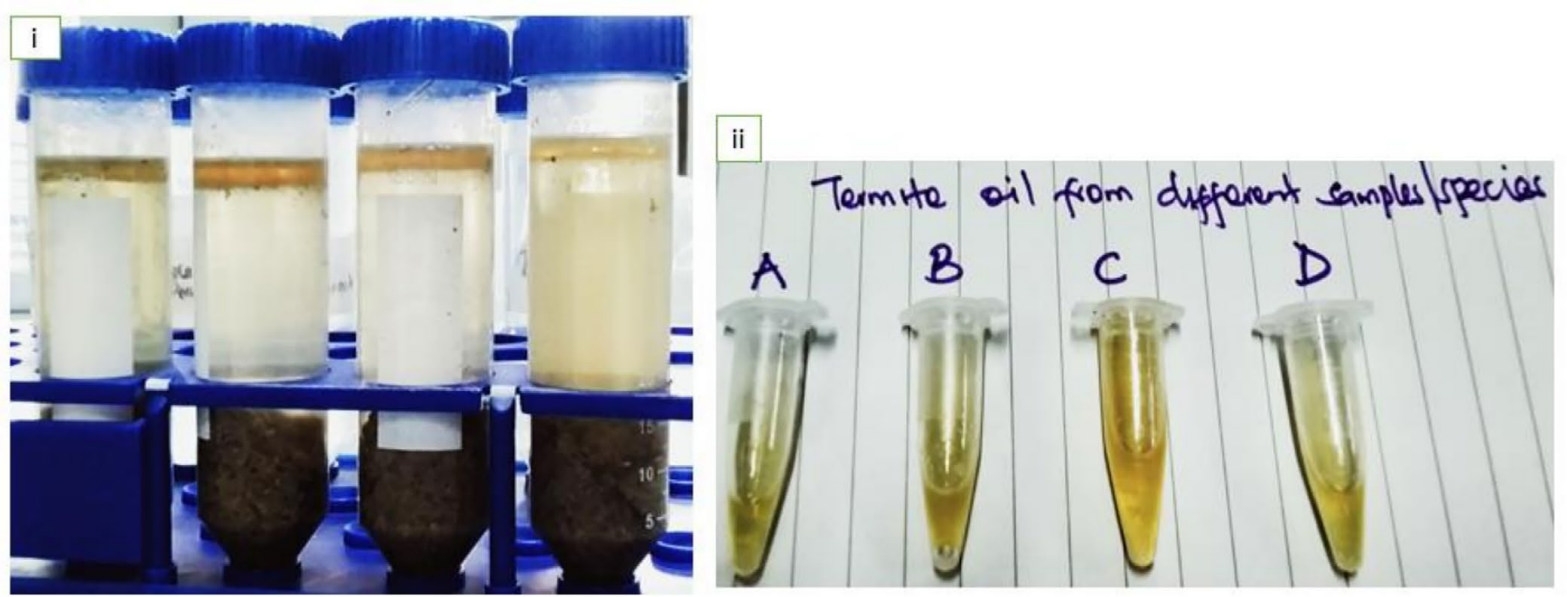
Ultrasound‐assisted termite oil extraction, with an ethanol‐isopropanol solvent; (i) Termite flour‐solvent suspensions upon settling, (ii) samples of recovered oil after sonication.

**FIGURE 2:**
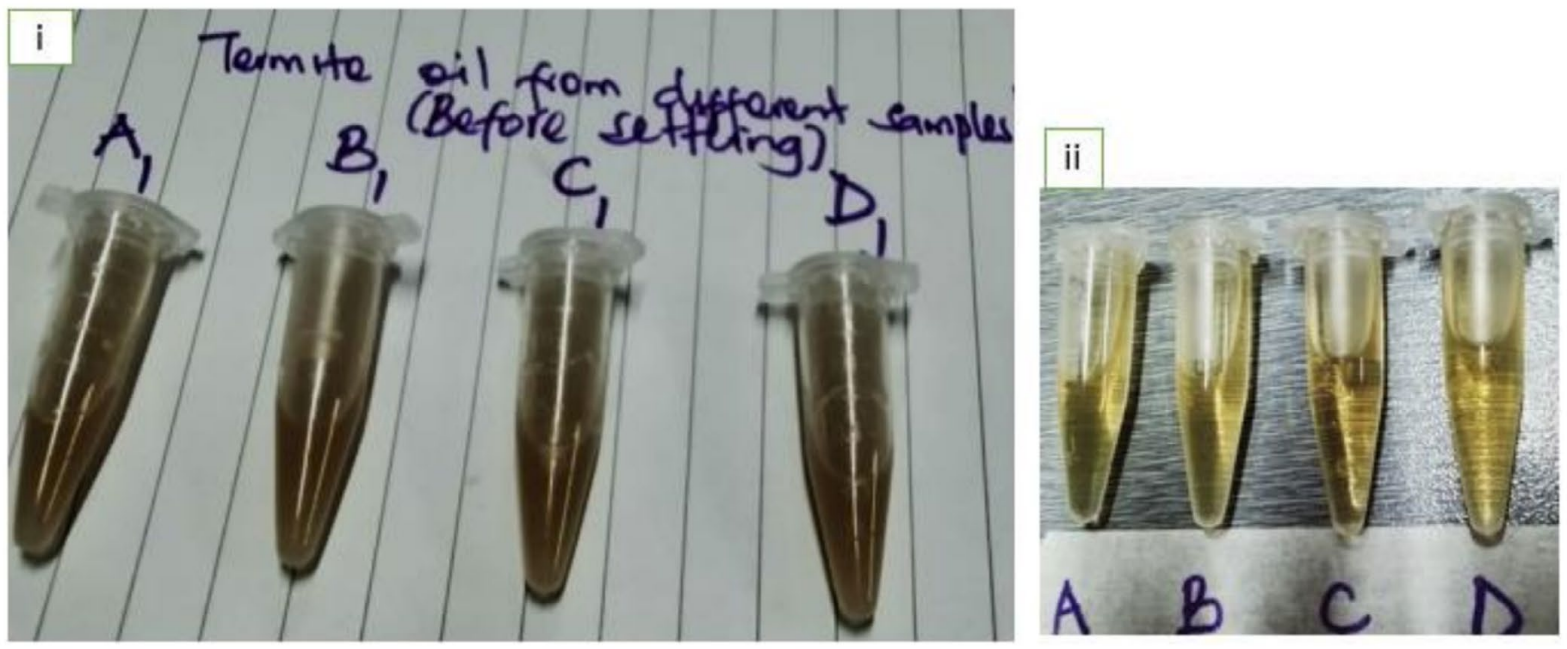
Extracted oil from edible winged termites' flour (mechanical press extraction, after which strained with a muslin cloth): (i) Before settling, (ii) Upper clearer layer collected off into separate tubes after settling.

- The conventional Soxhlet extraction method, which involved placing a thimble containing a 5 g termite flour sample inside a Soxhlet extractor (J.P. Selecta, S/No: 630086, Barcelona, Spain), and 20 mL of n‐hexane solvent (Merck, CAS No: 110‐54‐3) at 60°C, was poured into a pre‐weighed round‐bottomed flask/glass to extract crude fat from the sample, following the standard AOAC (2019) 991.36 method. The extraction duration was 8 h, followed by distillation to remove solvent from the extracted oil. The flask was then placed in a hot air oven (pol‐eko‐aparatura SP.J. S/No: SNISF170468, Poland) set at 100°C overnight to enable the solvent to evaporate and achieve sample concentration. The samples were then cooled in a desiccator before weighing. Crude fat was then determined using the following equation:

$$\%\mathrm{Fat}\;\text{content}=\frac{\text{Weight of glass with}\;\mathrm{fat}}{\text{Weight of}\;\mathrm{dry}\;\text{sample}}\times 100\%$$

ii Microwave‐assisted extraction, following the procedure of Hao et al. (2021) procedure with slight modifications: Briefly, 10 g of termite flour (moisture content of 5.13%–6.82% from our previous Khadijah et al. 2025) was placed in holders containing 200 mL n‐hexane as a solvent, and the mixture was shaken for 20 s before it was placed inside the microwave (Dawlance, Model: DW‐133G) for lipid extraction. The microwave was set at 260 W for an extraction duration of 30 min, and a solid‐to‐solvent ratio of 1:15 (w/v %) was used. This was followed by centrifuging the mixture (Rotofix 32A centrifuge, Ref 1206, S/No: 0032672–04, Germany) at room temperature for 20 min to facilitate separation of the liquid phase, after which a rotary evaporator (Model: EV311AD, 20‐80 rpm, S/N: 160117 V1480, LabTech Ltd.) was used for lipid recovery. The oil yield obtained was then calculated using the following equation:

$$\%\text{oil yield}=\frac{\text{Weight of extracted oil}}{\text{Weight of termite flour}}\times 100\%$$

iii Ultrasound‐assisted extraction following the Gharibzahedi and Altintas (2022) procedure, with slight modification: Briefly, 10 g of termite flour preheated in a hot air oven set at 150°C for 5 min was added to an ethanol‐isopropanol (1:1 v/v) solvent at a ratio of 22:5 v/w (solvent: termite flour ratio) in a 500 mL flask (Isopropanol, CAS: 67‐63‐0, VWR, USA). The resulting suspension was sonicated (Ultrasonicator Model VCX750, S/No: 131103AT‐10‐22, Sonics and Materials Inc. USA) at 20 kHz freq, power of 240 W, for 23 min in an ultrasonic bath (S/No: 100332076, Elmasonic E60H, Germany) filled with 75% distilled water and maintained at 70°C. The liquid in the flask was maintained at a lower level than that in the water bath. The temperature during oil extraction was continuously monitored with a thermometer and kept within the desired level by the addition of cold or hot water such that temperature fluctuations were only ±1°C. The amount of recovered oil (Figure 1) was then expressed as a percentage using the following equation:

$$\%\text{oil yield}=\frac{\text{Weight of extracted oil}}{\text{Weight of sample}\;\left(\text{flour}\right)}\times 100\%$$

iv Mechanical press extraction, which involved feeding termite flour into an oil press mill (Model YST‐DH80TB, China) set at 60°C to melt crude fat, aid fat separation, and facilitate the easy drainage of pressed oil after fat melting. By applying constant pressure to the machine, the oil was mechanically oozed from the flour. This was followed by straining the oil with muslin cloth and allowing it to settle. The upper layer was then collected in separate tubes (Figure 2) and quantified.

#### 2.1.2 Oil Characterization

Extracted termite oil was subjected to determination of oil color, solidification temperature, refractive index, total cholesterol content, saponification value, peroxide value, and iodine value. Values for oil color measurement, such as L* (lightness/whiteness), a* (green‐red intensity), b* (blue‐yellow intensity), C* (chromaticity for color saturation), and H* (hue angle for the color tone) of the samples were obtained using a precision colorimeter (NR110, S/No: 1107687, 3nh, China). Prior to the analysis, the oils were stored at 20°C for 10 min. The instrument was standardized during each sample measurement, and the mean of three readings from each sample for each color index of the scale (L*, a*, b*, C*, and H*) was recorded and analyzed. The solidification temperature was assessed using the open capillary method (AOAC 1996). The refractive index of termite oil was determined using a digital refractometer (Model DR301‐95, A. Kruss Optronic, Germany). Iodine, peroxide, and saponification values were analyzed according to the AOAC (1996) protocol.

The total cholesterol in termite flour samples was estimated using the Liebermann‐Buchard method, following the protocol used by Barreto (2005). Briefly, samples (0.5 mL) of the extracted termite oil were weighed into test tubes, followed by the addition of 5.0 mL of Liebermann‐Buchard reagent (Merck, US). Test tubes were vortexed (MX‐S vortex mixer, S/No: VB217AN0027040, Bioaquo, China) and then placed in a water bath (PolyScience, USA) at 35°C for 10 min, followed by the measurement of absorbance at 550 nm with a spectrophotometer (UV–visible double beam spectrophotometer, Model U‐2900, S/No: 29E01‐019, Hitachi, Japan). The total cholesterol content of the samples was quantified by comparing the absorbance to the standard.

### 2.2 Chitosan Extraction and Characterization

#### 2.2.1 Sample Preparation

Defatted termite flour samples (Soxhlet defatting procedure described in the previous section) were used for chitosan extraction. To ascertain that termite flour samples had been thoroughly defatted, representative samples were assessed for residual percentage fat content using the equation for Soxhlet analysis.

$$\%\mathrm{Fat}/\text{oil content}\;\left(\mathrm{dry}\;\text{weight}\right)=\frac{\text{Weight of glass with}\;\mathrm{fat}/\text{oil}}{\;\text{Weight of}\;\mathrm{dry}\;\text{sample}\;\left(\text{flour}\right)}\times 100$$

### 2.3 Chitosan Production

Chitosan production was performed using two techniques: (i) conventional chemical methods and (ii) green extraction technologies.

#### 2.3.1 Conventional Chemical Methods

These were performed following the procedures of Espinosa‐Solís et al. (2024) with slight modifications, and the following steps were employed:

- Demineralization (DM): Triplicates of 5 g of defatted termite flour samples were independently reacted with 2 M HCl (50 mL) at 30°C for 3 h. This was followed by filtering and rinsing the samples with hot distilled water until a pH of 7 was attained. The mixture was placed in an oven maintained at 60°C, followed by calcination in a muffle furnace (S/No: 0013152, Comecta, Barcelona, Spain) set at 900°C. The demineralization percentage (% DM) was assessed using the following formulae:

$$\%\mathrm{DM}=\frac{\text{Mineral content before treatment}–\text{Mineral content after treatment}}{\text{Mineral content before treatment}}\times 100$$

ii Deproteinization (DP): Demineralized flour (1.2 g) was reacted with 2.56 M NaOH (12 mL) at 80°C, and the mixture was left to stand for 45 min. Samples were then rinsed with hot distilled water until a pH of 8 was achieved. Consequently, the samples were placed in an oven set at 60°C overnight before protein assessment using the micro Kjeldahl method was carried out (Kjeldahl system: Behrotest Inkjel, Labor‐Technik, Germany). The percentage of deproteinization (% DP) was assessed by comparing the protein content before treatment with the protein content after treatment to confirm the success of protein removal using the equation below:

$$\%\mathrm{DP}=\frac{\text{Protein content before treatment}–\text{Protein content after treatment}}{\;\text{Protein content before treatment}}\times 100$$

Deproteinized termite flour was then oven‐dried at 60°C overnight.

iii Bleaching and purification: Samples of deproteinized flour weighing 5.65 g were mixed with 56.5 mL of 1% sodium hypochlorite solution (CAS: 7681‐52‐9, VWR, USA) in a flask and constantly stirred for 3 h at room temperature. This was followed by rinsing with distilled water and leaving the samples in an oven maintained at 60°C to dry overnight. The same procedure was repeated once for the samples to be bleached again so that purified chitin could be obtained.
iv Deacetylation (DA): Purified chitin weighing 5 g was mixed with 50% NaOH (200 mL), followed by autoclaving (Digital autoclave, 1280 × 960, Robus Technologies, UAE) the mixture at 121°C for three cycles of 45 min each. Upon completion of the reaction, the samples were rinsed with distilled water until a neutral pH was attained. The samples were left in an oven at 60°C to dry overnight. The resultant product was chitosan produced from termite flour, which was weighed (analytical balance: S/No: B552900980, Ohaus Corporation, USA) and expressed as a percentage using the equation below;

$$\text{Chitosan content}\;\left(\%\right)=\frac{\text{Extracted dried chitosan}\;\left(g\right)}{\;\mathrm{Raw}\;\text{material weight}\;\left(g\right)}\times 100$$

Dried chitosan samples were kept in airtight plastic bottles at 4°C, awaiting physico‐chemical characterization.

#### 2.3.2 Green Extraction Technologies

These were performed following Psarianos et al.'s (2022) procedures, as explained below:

- Independent Demineralization with *Lactococcus lactis* fermentation, citric acid, and microwave treatment. Lactic acid demineralization involved the fermentation of defatted termite flour with *L. lactis* (purchased and kept as a lyophilized culture). Sterile MRS broth (Cat No: DF0881‐17‐5, Fischer Scientific, Bioworld, USA) was mixed with the lyophilized culture and cultivated at 30°C for 48 h. This was followed by the addition of 10% w/v defatted termite flour, allowing fermentation to proceed at 30°C for 7 days with agitation at 150 rpm. Citric acid demineralization involved reacting defatted termite flour samples with 0.5 M citric acid (ratio 1:30) followed by agitation for 2 h at room temperature. Microwave‐assisted demineralization involved mixing defatted termite flour samples with a 1 M HCl solution (ratio 1:30), followed by microwave heating at 500 W for 8 min. The % DM for each method was calculated to assess the efficiency of each of the three demineralization processes.
- Deproteinization with bromelain treatment and papain enzyme digestion, using defatted termite flour previously demineralized with *L. lactis* fermentation (on the basis that no acid was involved in the demineralization, a purely green technique compared to other demineralization methods described above). Bromelain‐assisted deproteinization was performed by adding demineralized termite flour to distilled water (ratio 1:20), then digesting it through the bromelain enzyme treatment (Bromelain Enzyme BR, 300u/mg Cat # B834642, Macklin, China) with an enzyme/substrate ratio of 2% w/w for 5 h at 60°C after adjusting the pH value to 5.5, following guidelines provided by the commercial bromelain manufacturer. Papain‐assisted deproteinization involved adding demineralized flour to a 5 mM cysteine solution, which was digested through the enzymatic solution with an enzyme/substrate ratio of 1:100 (mg/mg). This was followed by adjusting the pH to 6.5 and then performing the process for 24 h at 60°C using the guidelines provided by the lyophilized papain powder manufacturer (CAS No: 9001‐73‐4, Merck). The % DP for each method was calculated to assess the efficiency of each deproteinization process.

Each deproteinization process was followed by drying of the sample, bleaching, and purification, followed by deacetylation and weighing of the chitosan produced following the same procedures used during conventional chemical extraction.

### 2.4 Chitosan Characterization

Physico‐chemical characterization involved assessing chitosans' water binding capacity, molecular weight, and fat binding capacity, following the procedures used by Luo et al. (2019), with slight modifications. Briefly, the water binding capacity was assessed by weighing chitosan (0.5 g) into a centrifuge tube, mixing it with 10 mL of distilled water, and leaving the mixture at room temperature for a total period of 30 min. The mixture was vortexed for 5 s every 10 min. The mixture was centrifuged at 3500 rpm for 25 min, the supernatant was discarded, and the contents of the tube were weighed. The water‐binding capacity was then determined using the following equation:

$$\text{Water}-\text{binding capacity}\;\left(\%\right)=\frac{\text{Water bound}\;\left(g\right)}{\;\text{Initial chitosan weight}\;\left(g\right)}\times 100$$

To assess the fat‐binding capacity, chitosan (0.5 g) was mixed with 10 mL soybean oil in a centrifuge tube, and the tube was placed at room temperature. Vortex shaking was performed for 5 s every 10 min, until a total of 30 min period had elapsed. This was followed by centrifugation at 3500 rpm for 25 min, discarding the supernatant oil and weighing the centrifuge tube with its contents. The fat‐binding capacity was then assessed using the equation below:

$$\mathrm{Fat}-\text{binding capacity}\;\left(\%\right)=\frac{\mathrm{Fat}\;\text{bound}\;\left(g\right)}{\;\text{Initial chitosan weight}\;\left(g\right)}\times 100$$

Viscometry method was employed to determine the molecular weight of the extracted chitosan; chitosan solutions were prepared by stirring the produced chitosan with 0.5 M acetic acid and 0.5 M sodium acetate buffer at 25°C for 24 h at room temperature, then measuring the viscosity of the resultant solution. The molecular weight of chitosan was estimated using the Mark–Houwink–Sakurada equation below:

$$\left[\eta \right]=K\;{\left[\mathrm{Mw}\right]}^{a}$$

where K (0.119) and a (0.59) are constants, η is intrinsic viscosity, and Mw is molecular weight.

### 2.5 In Vitro Assessment of the Extracted Chitosan's Antimicrobial Ability

This was performed by employing the procedure used by Lagat et al. (2021) with slight changes, as detailed in the preceding sections.

#### 2.5.1 Test Organisms for the Antimicrobial Assay

Instead of using more than one microbe from a single group, the present study focused on a microorganism from each of the three common groups of pathogenic microbes: a gram‐positive bacterium ( *Staphylococcus aureus* ), a gram‐negative bacterium ( *Escherichia coli* ), and an opportunistic pathogenic yeast ( *Candida albicans* ). The idea was that each of the above pathogenic microbes would be representative of the antimicrobial behavior likely to be displayed by other microbe members of that same group. These microbes were commercially purchased from approved suppliers. Lyophilized pellets of *S. aureus* ATCC 25923 and *E. coli* ATCC 8739 were purchased from Microbiologics (0827P Cat No: 23‐003‐3513 and 0483E3 Cat No: 23‐003‐5004, Thermo Fischer Scientific), whereas *C. albicans* ATCC 10231 was purchased from MicroBioLogics 0443P (Cat No: 23‐021155, Thermo Fischer Scientific).

#### 2.5.2 Inoculum Preparation

Each of the two bacterial strains was sub‐cultured overnight in nutrient broth (Sigma‐Aldrich, USA) maintained at 37°C, whereas *C. albicans* was sub‐cultured overnight at the same temperature in Sabouraud dextrose broth (Sigma‐Aldrich, USA) and then on agar plates. This was followed by suspending bacterial colonies in sterile saline solution (9 mL), and the suspension was adjusted to achieve turbidity equivalent to a standardized inoculum of 1 × 10^8^ colony forming units (CFUs)/mL with McFarland standards streaked onto the surface of sterile agar plates (0.5 mL). Mueller–Hinton agar (Lot L20014005LCMB, BioPLUS Chemicals, UK) was then prepared, and 12 mL of the medium was dispensed into petri dishes and allowed to solidify.

#### 2.5.3 Preparation of Chitosan Solutions

In this antimicrobial experiment, the positive control was commercial chitosan from crustacean shells (Sigma‐Aldrich, USA), whereas double‐distilled water was used as the negative control. Dried samples of termite chitosan and commercial chitosan were independently dissolved in a solvent, 1% acetic acid (Sigma‐Aldrich, USA). After stirring, the solutions were filtered through Whatman No. 42 filter paper (Cat No: 1442125, England) to eliminate impurities before being stored at 4°C. Some studies have reported antimicrobial properties of acetic acid, depending on the concentration used (Guarnieri et al. 2022), and thus, to separate the antimicrobial activity of termite chitosan from that of its solvent (acetic acid), the present study also assessed acetic acid alone (2 mL of 1% acetic acid) without any chitosan.

#### 2.5.4 Antimicrobial Susceptibility Assay

The Kirby–Bauer disc diffusion method was used, following the procedure of Kourmouli et al. (2018), with slight modifications, as detailed below. Twenty microliters of freshly prepared cultures of *S. aureus, E. coli*, and *C. albicans* were inoculated and spread uniformly onto Mueller–Hinton agar plates. Fifty microliters of chitosan solution were impregnated onto sterile filter paper discs to form chitosan sample discs. This was followed by air‐drying the chitosan sample discs and placing them on top of the agar plates. The plates were then incubated at 37°C for 24 h using an incubator (S/No: 15‐20062, Binder GmbH, Germany). The presence of clear inhibition zones on the discs was considered to be evidence of antimicrobial activity. On each plate, commercial chitosan, double‐distilled water, and acetic acid alone were used at the same concentrations as the termite chitosan solutions (50 μL). Experiments for each test organism were performed in triplicate. Antimicrobial activity was assessed by measuring the diameter (in mm) of clear zones of inhibition around each disc on each plate of each test microbe, and the mean diameters were obtained from three independent biological replicates.

### 2.6 Statistical Analysis

Data for the present study were processed using SAS software (version 9.4, SAS Institute Inc., Cary, NC, USA). Analysis of variance (ANOVA) was conducted to determine significant differences, and means were separated using the Tukey post hoc test. Each experiment was done in triplicate, and all statistical analyses were considered statistically significant at *p < 0.05*.

## 3 Results

In the result and discussion section, the word ‘significant difference’ wherever used implied that the *p*‐value was less than 0.05.

### 3.1 Comparison of the Oil Yield From Different Termite Species and Extraction Techniques

The oil yields from the four edible winged termite flours extracted using four different techniques are given in Table 1. Mechanical pressing yielded the significantly (*p* < 0.001) highest oil quantity (37.42%–38.50%) among all the species, followed by microwave‐assisted extraction. Overall, the significantly lowest oil yield was observed in the Soxhlet/conventional extraction method (8.05%–18.92%).

**TABLE 1:** Comparison of oil yield from four different species and extraction techniques.

Termite species	Oil yield (%)			
	Soxhlet extraction	Microwave‐assisted extraction	Ultrasound‐assisted extraction	Mechanical pressing
*M. subhyalinus*	18.60 ± 0.64^2,a^	24.91 ± 0.06^2,a^	22.66 ± 0.15^2,a^	37.42 ± 0.33^1,a^
*M. bellicosus*	18.92 ± 0.33^3,a^	29.04 ± 0.52^2,a^	21.47 ± 0.48^3,a^	38.18 ± 0.71^1,a^
*P. spriniger*	13.25 ± 0.46^4,ab^	28.85 ± 0.41^2,a^	21.95 ± 0.33^3,a^	38.50 ± 0.00^1,a^
*O. lateritius*	8.05 ± 0.37^4,b^	29.27 ± 0.69^2,a^	20.83 ± 0.27^3,a^	37.69 ± 0.44^1,a^

*Note:* Values in the table are Mean ± Standard deviation of 3 replicates. Values with the same superscript figure in the same row (comparison among extraction techniques) are not significantly different at *p* < 0.05, whereas values with the same superscript letter in the same column (comparison among termite species) are not significantly different at *p* < 0.05, using Tukey test (Two‐way ANOVA).

Upon assessment of termite species differences for each extraction method, microwave‐assisted extraction, mechanical pressing, and ultrasound‐assisted extraction did not show species differences in oil yield. On the contrary, species differences were evident in soxhlet extraction, where *O. lateritius* produced the significantly lowest oil yield (8.05%), whereas *M. subhyalinus* and *M. bellicosus* produced the significantly highest oil yield, that is, 18.60%–18.92% (no significant differences existed between the oil yield in these two termite species). For the current study, therefore, the differences in termite species did not influence the oil yield when green extraction techniques were used. However, the oil yield in the conventional Soxhlet extraction technique was affected by species.

### 3.2 Characterization of the Extracted Oil

Generally, the extracted termite oils appeared as clear, golden‐to‐light yellow liquids (Figure 1, ii). However, mechanically pressed oil appeared brownish (Figure 2, ii), and presented the lowest L* and b* values ranging from 34.72 to 35.47 and 30.62% to 34.04%, respectively, in comparison with termite oils extracted by other methods (Table 2). It was observed that termite oil produced by ultrasound‐assisted extraction produced the lightest and least brownish color (significantly higher L* and b* values; *p* < 0.007), indicating a whiter tone, followed by microwave‐assisted extraction. For each oil extraction method, no species differences were significant in the yellowness index of the oil; that is, all the termite species showed significantly similar b* values. The findings of the current study exhibited haphazard variation with no significant differences in the green‐red intensity of oil (a* index) among both the termite species and oil extraction techniques. It was also observed that all the extracted termite oils produced a very low redness index; that is, a* value which ranged from 0.85 to 0.91. The chromaticity index (C*) of the extracted termite oil ranged from 8.07 to 13.41, whereas the hue angle (H*) ranged from 74.33 to 85.26, and no significant differences were observed between the extraction techniques and termite species. Therefore, in the present study, oil color characteristics were not influenced by termite species.

**TABLE 2:** Characterization of termite oil from different species and extraction techniques.

Physicochemical characteristics and the total cholesterol content			Termite species			
			*M. subhyalinus*	*M. bellicosus*	*P. spriniger*	*O. lateritius*
Oil color	L*	Soxhlet‐extracted	57.49 ± 0.26^b,1^	57.13 ± 0.02^b,1^	57.40 ± 0.11^b,1^	57.38 ± 0.50^b,1^
		Microwave‐assisted	68.36 ± 0.54^a,1^	68.05 ± 0.88^a,1^	69.14 ± 0.70^a,1^	68.03 ± 0.21^a,1^
		Ultrasound‐assisted	73.46 ± 0.93^a,1^	72.34 ± 0.07^a,1^	72.20 ± 0.41^a,1^	71.97 ± 0.69^a,1^
		Mechanical pressing	35.47 ± 0.39^c,1^	34.72 ± 0.82^c,1^	35.12 ± 0.14^c,1^	34.94 ± 0.38^c,1^
	a*	Soxhlet‐extracted	0.91 ± 0.49^a,1^	0.87 ± 0.06^a,1^	0.90 ± 0.32^a,1^	0.86 ± 0.51^a,1^
		Microwave‐assisted	0.88 ± 0.11^a,1^	0.91 ± 0.77^a,1^	0.85 ± 0.16^a,1^	0.90 ± 0.02^a,1^
		Ultrasound‐assisted	0.89 ± 0.62^a,1^	0.86 ± 0.33^a,1^	0.90 ± 0.94^a,1^	0.87 ± 0.10^a,1^
		Mechanical pressing	0.91 ± 0.35^a,1^	0.85 ± 0.00^a,1^	0.86 ± 0.72^a,1^	0.91 ± 0.44^a,1^
	b*	Soxhlet‐extracted	55.81 ± 0.06^b,1^	54.52 ± 0.28^b,1^	56.00 ± 0.63^b,1^	54.74 ± 0.06^b,1^
		Microwave‐assisted	55.32 ± 0.67^b,1^	50.19 ± 0.07^b,1^	53.51 ± 0.42^b,1^	55.90 ± 0.31^b,1^
		Ultrasound‐assisted	68.48 ± 0.80^a,1^	66.09 ± 0.52^a,1^	67.30 ± 0.14^a,1^	66.90 ± 0.55^a,1^
		Mechanical pressing	32.33 ± 0.16^c,1^	34.04 ± 0.50^c,1^	30.62 ± 0.06^c,1^	33.28 ± 0.28^c,1^
	C*	Soxhlet‐extracted	9.30 ± 0.82^a,1^	8.64 ± 0.30^a,1^	9.05 ± 0.47^a,1^	8.07 ± 0.53^a,1^
		Microwave‐assisted	11.92 ± 0.57^a,1^	11.85 ± 0.64^a,1^	11.47 ± 0.28^a,1^	12.10 ± 0.97^a,1^
		Ultrasound‐assisted	13.41 ± 0.24^a,1^	12.54 ± 0.57^a,1^	12.73 ± 0.13^a,1^	13.15 ± 0.15^a,1^
		Mechanical pressing	8.75 ± 0.60^a,1^	8.61 ± 0.22^a,1^	9.08 ± 0.16^a,1^	8.82 ± 0.43^a,1^
	H*	Soxhlet‐extracted	74.33 ± 0.39^a,1^	75.55 ± 0.08^a,1^	74.86 ± 0.37^a,1^	75.12 ± 0.86^a,1^
		Microwave‐assisted	84.21 ± 0.61^a,1^	80.85 ± 0.03^a,1^	85.04 ± 0.00^a,1^	83.05 ± 0.29^a,1^
		Ultrasound‐assisted	83.95 ± 0.02^a,1^	82.62 ± 0.55^a,1^	85.26 ± 0.73^a,1^	81.56 ± 0.80^a,1^
		Mechanical pressing	76.68 ± 0.40^a,1^	77.19 ± 0.03^a,1^	77.43 ± 0.00^a,1^	79.25 ± 0.81^a,1^
Solidification temperature (°C)		Soxhlet‐extracted	11.40 ± 0.25^a,1^	12.10 ± 0.06^a,1^	12.60 ± 0.47^a,1^	12.90 ± 0.98^a,1^
		Microwave‐assisted	10.50 ± 0.47^a,1^	11.04 ± 0.62^a,1^	10.70 ± 0.61^a,1^	10.10 ± 0.45^a,1^
		Ultrasound‐assisted	10.90 ± 0.35^a,1^	10.30 ± 0.62^a,1^	10.40 ± 0.19^a,1^	11.00 ± 0.77^a,1^
		Mechanical pressing	11.30 ± 0.46^a,1^	11.10 ± 0.15^a,1^	11.50 ± 0.21^a,1^	11.70 ± 0.39^a,1^
Refractive index (nD)		Soxhlet‐extracted	1.23 ± 0.02^a,1^	1.35 ± 0.27^a,1^	1.31 ± 0.31^a,1^	1.27 ± 0.73^a,1^
		Microwave‐assisted	1.46 ± 0.02^a,1^	1.43 ± 0.66^a,1^	1.44 ± 0.91^a,1^	1.46 ± 0.15^a,1^
		Ultrasound‐assisted	1.43 ± 0.84^a,1^	1.40 ± 0.32^a,1^	1.44 ± 0.70^a,1^	1.41 ± 0.25^a,1^
		Mechanical pressing	1.39 ± 0.25^a,1^	1.39 ± 0.98^a,1^	1.34 ± 0.06^a,1^	1.37 ± 0.17^a,1^
Iodine value (g iodine/100 g oil)		Soxhlet‐extracted	104.00 ± 0.19^b,1^	105.00 ± 0.07^b,1^	103.00 ± 0.16^b,1^	108.00 ± 0.22^a,1^
		Microwave‐assisted	100.00 ± 0.52^b,2^	107.00 ± 0.00^b,1^	101.00 ± 0.50^b,2^	110.00 ± 0.11^a,1^
		Ultrasound‐assisted	81.00 ± 0.52^c,1^	82.00 ± 0.88^c,1^	80.00 ± 0.01^c,1^	83.00 ± 0.74^b,1^
		Mechanical pressing	117.00 ± 0.69^a,1^	121.00 ± 0.92^a,1^	119.00 ± 0.46^a,1^	111.00 ± 0.72^a,2^
Peroxide value (mg KOH/g)		Soxhlet‐extracted	0.75 ± 0.04^a,1^	0.79 ± 0.11^a,1^	0.69 ± 0.23^a,1^	0.73 ± 0.05^a,1^
		Microwave‐assisted	0.31 ± 0.82^a,1^	0.33 ± 0.19^a,1^	0.34 ± 0.74^a,1^	0.30 ± 0.52^a,1^
		Ultrasound‐assisted	0.25 ± 0.55^a,1^	0.27 ± 0.30^a,1^	0.23 ± 0.28^a,1^	0.26 ± 0.39^a,1^
		Mechanical pressing	0.19 ± 0.63^a,1^	0.18 ± 0.29^a,1^	0.17 ± 0.05^a,1^	0.18 ± 0.42^a,1^
Saponification value (mg KOH/g)		Soxhlet‐extracted	109.34 ± 0.61^a,1^	108.01 ± 0.88^a,1^	103.60 ± 0.27^b,2^	104.15 ± 0.40^b,2^
		Microwave‐assisted	107.00 ± 0.94^a,1^	101.25 ± 0.07^b,2^	101.81 ± 0.40^b,2^	110.01 ± 0.72^a,1^
		Ultrasound‐assisted	109.41 ± 0.53^a,1^	103.07 ± 0.26^b,2^	102.55 ± 0.29^b,2^	101.93 ± 0.65^b,2^
		Mechanical pressing	111.07 ± 0.73^a,1^	110.41 ± 0.44^a,1^	113.92 ± 0.18^a,1^	112.50 ± 0.47^a,1^
Total cholesterol content (mg/100 mL)		Soxhlet‐extracted	33.21 ± 0.85^a,2^	39.00 ± 0.42^a,1^	34.15 ± 0.60^a,2^	32.08 ± 0.03^a,2^
		Microwave‐assisted	32.95 ± 0.71^a,2^	38.52 ± 0.75^a,1^	32.84 ± 0.09^a,2^	31.0.21 ± 0.85^a,2^
		Ultrasound‐assisted	24.98 ± 0.31^b,1^	27.00 ± 0.59^b,1^	25.41 ± 0.61^b,1^	25.58 ± 0.70^b,1^
		Mechanical pressing	32.66 ± 0.47^a,1^	32.95 ± 0.26^ab,1^	31.10 ± 0.48^a,1^	30.73 ± 0.92^a,1^

*Note:* Values in the table are Mean ± SD from triplicate determination. Different superscript letters in the same column (comparison among extraction techniques) indicate significant differences (*p* < 0.05), whereas different superscript figures in the same row (comparison among termite species) indicate significant differences at *p* < 0.05, using Tukey test (Two‐way ANOVA).

The oil solidification temperature ranged between 10.1°C and 12.9°C. Although no significant differences were observed, Soxhlet‐extracted oil showed the highest solidification temperature, followed by mechanical pressing. The refractive index of termite oil ranged from 1.23 to 1.46 and was highest in microwave‐extracted oil, followed by ultrasound‐assisted extracted oil. The extraction techniques and termite species used did not show any significant differences. Mechanically pressed oil produced the highest iodine value (111–121 g iodine/100 g oil), whereas oil produced via ultrasound‐assisted extraction yielded the lowest iodine value. Differences in termite species were observed between the microwave‐extracted oil and mechanically pressed oil. The peroxide value of termite oil ranged from 0.17 to 0.79 mg KOH/g. Generally, Soxhlet‐extracted oil produced the highest peroxide value, whereas mechanically pressed oil produced the lowest value. The peroxide values did not show significant differences between the oil extraction techniques and termite species. Mechanically pressed oils had the highest saponification value (110.41–113.92 mg KOH/g). Oil from *M. subhyalinus* had a significantly higher saponification value than oils from other termite species.

The total cholesterol content of termite oils ranged from 24.98 to 39.00 mg/100 mL. Ultrasound‐assisted oil extraction yielded the significantly lowest total cholesterol content (24.98–27.00 mg/100 mL). The highest total cholesterol content was observed in oils extracted using Soxhlet and microwave‐assisted extraction methods. Oil from almost all termite species had significantly similar total cholesterol content, except *M. bellicosus* oil, which contained significantly higher total cholesterol content (up to 39.00 mg/100 mL).

### 3.3 Chitosan Extraction From Different Techniques and Species

Results presented in Table 3 indicate that the fermentation method yielded the significantly (*p < 0.003*) highest demineralization percentage in all the termite species (93.88%–96.06%), whereas citric acid treatment showed the lowest demineralization percentage (70.42%–76.83%). The demineralization percentage did not show a significant difference between the chemical method and microwave treatment. The chemical method produced the significantly highest deproteinization percentage (40.77%–43.71%) and chitosan yield (6.05%–7.00%) among the two enzymatic treatments assessed. No significant differences were observed in the deproteinization percentage between bromelain and papain enzyme treatments. However, papain enzyme treatment produced a significantly higher chitosan yield (4.61%–5.89%) than bromelain enzyme treatment (2.30%–4.20%). *M. bellicosus* produced a significantly higher chitosan yield than other termite species.

**TABLE 3:** Comparison of the efficiency and yield among different chitosan extraction techniques from different termite species.

Efficiency and chitosan yield from different extraction techniques		Termite species			
		*M. subhyalinus*	*M. bellicosus*	*P. spriniger*	*O. lateritius*
Demineralisation %	Chemical method	80.73 ± 0.84^b,1^	83.12 ± 0.65^b,1^	81.88 ± 0.02^b,1^	80.05 ± 0.47^b,1^
	Fermentation	94.27 ± 0.71^a,1^	93.88 ± 0.94^a,1^	96.06 ± 0.37^a,1^	95.57 ± 0.28^a,1^
	Citric acid treatment	73.74 ± 0.56^c,1^	70.42 ± 0.11^c,1^	76.83 ± 0.06^c,1^	72.10 ± 0.32^c,1^
	Microwave treatment	83.60 ± 0.59^b,1^	80.73 ± 0.22^b,1^	84.01 ± 0.13^b,1^	82.91 ± 0.75^b,1^
Deproteinization %	Chemical method	43.71 ± 0.31^a,1^	42.59 ± 0.66^a,1^	40.77 ± 0.08^a,1^	43.22 ± 0.69^a,1^
	Bromelain treatment	21.05 ± 0.00^b,1^	20.47 ± 0.98^b,1^	26.60 ± 0.54^b,1^	23.81 ± 0.76^b,1^
	Papain treatment	25.96 ± 0.71^b,1^	24.35 ± 0.40^b,1^	22.87 ± 0.15^b,1^	27.23 ± 0.49^b,1^
Chitosan yield (%)	Chemical method	6.05 ± 0.49^a,1^	6.20 ± 0.31^a,1^	7.00 ± 0.59^a,1^	6.94 ± 0.88^a,1^
	Bromelain treatment	4.20 ± 0.15^b,1^	4.19 ± 0.10^b,1^	2.30 ± 0.07^c,3^	3.17 ± 0.90^c,2^
	Papain treatment	4.61 ± 0.53^b,2^	5.89 ± 0.01^a,1^	4.77 ± 0.32^b,2^	5.00 ± 0.10^b,1^

*Note:* Values in the table are Mean ± Standard deviation of 3 replicates. Values with the same superscript letter in the same column (comparison among extraction techniques) are not significantly different at *p* < 0.05, whereas values with the same superscript figure in the same row (comparison among termite species) are not significantly different at *p* < 0.05 using Tukey test (Two‐way ANOVA).

### 3.4 Characterization of the Extracted Chitosan

The molecular weight of the extracted chitosan varied from 198.2 × 10^3^ to 366 × 10^3^ g/Mole (Table 4). In all termite species, enzymatically extracted chitosan produced significantly (*p* < 0.0001) higher molecular weights than chemically extracted chitosan. The molecular weight did not show significant differences between bromelain‐ and papain‐extracted chitosan. Species differences were manifested only in *P. spriniger*, which generally produced the lowest molecular weight, that is, (198.2–338.4) × 10^3^ g/Mole.

**TABLE 4:** Physico‐chemical characterization of the extracted termite chitosan.

Physico‐chemical characteristics of termite chitosan		Termite species			
		*M. subhyalinus*	*M. bellicosus*	*P. spriniger*	*O. lateritius*
Molar mass × 10^3^ (g/Mole)	Chemically‐extracted	271.20 ± 0.83^b,1^	247.00 ± 0.65^b,1^	198.20 ± 0.44^b,2^	250.70 ± 0.31^b,1^
	Bromelain‐extracted	340.70 ± 0.52^a,1^	358.20 ± 0.01^a,1^	334.60 ± 0.52^a,1^	341.00 ± 0.70^a,1^
	Papain‐extracted	366.00 ± 0.91^a,1^	360.50 ± 0.72^a,1^	338.40 ± 0.09^a,2^	355.80 ± 0.12^a,1^
Water‐binding capacity (%)	Chemically‐extracted	568.00 ± 0.69^a,1^	565.00 ± 0.11^a,1^	572.00 ± 0.20^a,1^	567.00 ± 0.76^a,1^
	Bromelain‐extracted	251.00 ± 0.60^c,1^	254.00 ± 0.09^c,1^	259.00 ± 0.31^b,1^	255.00 ± 0.40^c,1^
	Papain‐extracted	317.00 ± 0.82^b,1^	305.00 ± 0.74^b,1^	279.00 ± 0.00^b,2^	314.00 ± 0.59^b,1^
Fat‐binding capacity (%)	Chemically‐extracted	257.00 ± 0.52^a,1^	238.00 ± 0.01^a,1^	247.00 ± 0.90^a,1^	241.00 ± 0.53^a,1^
	Bromelain‐extracted	244.00 ± 0.48^a,1^	231.00 ± 0.63^a,1^	207.00 ± 0.57^b,2^	236.00 ± 0.85^a,1^
	Papain‐extracted	128.00 ± 0.74^b,2^	149.00 ± 0.81^b,1^	124.00 ± 0.53^c,2^	121.00 ± 0.90^b,2^

*Note:* Values in the table are Mean ± Standard deviation of 3 replicates. Values with the same superscript letter in the same column (comparison of chitosan extracted with different techniques) are not significantly different at *p* < 0.05, whereas values with the same superscript figure in the same row (comparison among termite species) are not significantly different at *p* < 0.05, using Tukey test (Two‐way ANOVA).

In all the termite species, water and fat binding capacities were significantly highest in chemically‐extracted chitosan, that is, 565%–572% and 238%–257%, respectively. Bromelain‐extracted chitosan showed the lowest water binding capacity, whereas papain‐extracted chitosan had the lowest fat binding capacity.

### 3.5 In Vitro Assessment of the Extracted Chitosan's Antimicrobial Ability

All chitosan samples produced from edible winged termite flours influenced the development of measurable microbial suppression zones against both bacteria (gram‐positive and gram‐negative) and yeast (Table 5). However, varying degrees of antimicrobial activity were observed against *S. aureus* , *E. coli* , and *C. albicans* . Except for *M. subhyalinus*, chitosan from the other three termite species generally showed significantly highest antimicrobial activity against *E. coli* followed by *S. aureus* , whereas the significantly lowest antimicrobial activity was observed against *C. albicans* . Chitosan from *M. subhyalinus* did not show a significant difference in antimicrobial activity between *S. aureus* and *E. coli* . In most termite species, chemically extracted chitosan exhibited significantly higher antimicrobial activity than the two enzymatically extracted chitosans (bromelain and papain‐extracted chitosan), and this effect was most pronounced in *S. aureus* and *E. coli* . In all the termite species, no significant differences existed in antimicrobial activity between bromelain and papain‐extracted chitosan. Commercial chitosan from crustacean shells showed very high antimicrobial activity against all tested microbes. The antimicrobial activity of commercial chitosan was almost comparable to that of chemically extracted termite chitosan against *E. coli* for most termite species. Acetic acid alone (without chitosan) formed very small, that is, almost negligible microbial inhibition zones (1.38 ± 0.45 and 2.53 ± 0.91 mm), except in *E. coli* where significantly higher and slightly considerable antimicrobial activity, that is, (6.44 ± 0.70 mm) was observed. As expected, distilled water, the negative control used, did not show any microbial inhibition zone.

**TABLE 5:** Antimicrobial ability of the extracted termite chitosan against specific pathogenic microorganisms.

Termite species	Chitosan extraction method	Diameter for the clear zones of inhibition around the disc on the microbial plate (mm)		
		*S. aureus*	*E. coli*	*C. albicans* (Yeast)
*M. subhyalinus*	Chemically‐extracted	18.94 ± 0.61^a,1^	19.03 ± 0.47^a,1^	7.25 ± 0.33^b,1^
	Bromelain‐extracted	11.85 ± 0.74^a,2^	13.49 ± 0.81^a,2^	6.44 ± 0.53^b,1^
	Papain‐extracted	11.93 ± 0.06^a,2^	14.00 ± 0.50^a,2^	7.21 ± 0.95^b,1^
*M. bellicosus*	Chemically‐extracted	12.61 ± 0.55^b,1^	20.71 ± 0.42^a,1^	10.53 ± 0.82^b,1^
	Bromelain‐extracted	10.82 ± 0.81^a,1^	13.69 ± 0.51^a,2^	6.00 ± 0.79^b,2^
	Papain‐extracted	10.08 ± 0.05^b,1^	14.93 ± 0.02^a,2^	6.27 ± 0.31^b,2^
*P. spriniger*	Chemically‐extracted	22.97 ± 0.48^a,1^	25.19 ± 0.63^a,1^	10.90 ± 0.57^b,1^
	Bromelain‐extracted	9.42 ± 0.77^b,2^	17.38 ± 0.14^a,2^	8.21 ± 0.85^b,1^
	Papain‐extracted	10.15 ± 0.13^b,2^	17.52 ± 0.75^a,2^	7.89 ± 0.03^b,1^
*O. lateritius*	Chemically‐extracted	22.83 ± 0.00^a,1^	23.76 ± 0.50^a,1^	11.41 ± 0.11^b,1^
	Bromelain‐extracted	10.77 ± 0.61^b,2^	16.71 ± 0.45^a,2^	6.03 ± 0.52^c,2^
	Papain‐extracted	7.55 ± 0.35^b,2^	17.04 ± 0.62^a,2^	6.78 ± 0.39^b,2^
***p***		**< 0.005**	**< 0.009**	**< 0.001**
***F***		**2.270**	**1.641**	**3.050**
Commercial chitosan (+ve control)		23.04 ± 0.15^a^	24.31 ± 0.46^a^	22.92 ± 0.11^a^
1% acetic acid (solvent used) without chitosan		2.53 ± 0.91^b^	6.44 ± 0.70^a^	1.38 ± 0.45^b^
Double distilled water (−ve control)		0.00 ± 0.00^a^	0.00 ± 0.00^a^	0.00 ± 0.00^a^

*Note:* Values in the table are Mean ± Standard deviation of 3 replicates. Values with different superscript letters (a, b, c) in the same row (comparison of antimicrobial activity among test microbes) indicate significant differences at *p* < 0.05, whereas values with different superscript figures (1, 2) in the same column for a particular termite species (comparison of extraction methods) indicate significant differences at *p* < 0.05. Tukey test was used to separate means where significant differences existed.

## 4 Discussion

### 4.1 Comparison of the Oil Yield From Different Species and Extraction Techniques

Recently, research aimed at extracting insect oils/lipids for use as food ingredients has increased. According to Antunes et al. (2024), insect oils used as food ingredients can enhance consumer acceptance of edible insects, especially in countries/regions that still grapple with entomophagy neophobia. Edible‐winged termites are naturally rich in oil, which explains why they are fried in their own oil during processing (Khadijah et al. 2024; Kinyuru 2020; Siddiqui et al. 2023). Except in Kinyuru (2020, 2009), Ajayi (2012), Ekpo and Onigbinde (2007) theses, no other studies could be traced regarding the extraction and characterization of termite oil yet its potential as a ‘healthier oil for humans than most animal and plant oils’ is well documented. From our previous study (Khadijah et al. 2025), complete fatty acid analysis showed that the investigated termite flours had more types of unsaturated fatty acids (08) than the saturated ones (04), with oleic acid (C18:1 ω‐9)‐a monounsaturated fatty acid yielding the highest quantity of fatty acids (35.36%–44.78%) and *M. bellicosus* plus *P. spriniger* showing the significantly highest quantity. This makes edible‐winged termites a healthier food choice than livestock‐based sources, as explained further in the proceeding section on ‘characterization of the extracted oil, especially the subsection for Total cholesterol content’. The present study provides evidence that, similar to virgin oils (e.g., linseed oil, arachis oil, olive oil, etc.), termite oil can be extracted physically by pressing under minimal heat, without the need for chemical treatment (Figure 2). Evidence also showed that when ultrasonication was used, the oil might not need to be refined (Figure 1). To date, the available literature on the extraction of oil from edible‐winged termites has not reported inclusion of a refining step (Khadijah et al. 2024). According to Rahman et al. (2024), the oil recovered from the screw press after mechanical extraction must be refined. In the present study, however, mechanically extracted oil was not refined; the upper oil layer collected into separate tubes after separation appeared clear (Figure 2).

Various extraction methods have been employed, such as Soxhlet, supercritical CO_2_, Folch, aqueous extraction, and mechanical pressing, to extract insect lipids for edible use (Purschke et al. 2017; Rahman et al. 2024; Tzompa‐Sosa et al. 2014; van Huis and Rumpold 2023). Rahman et al. (2024) and Tzompa‐Sosa et al. (2014) highlighted that the extraction method affects the quantity of the extracted insect oils. Conventional and green extraction techniques, that is, Soxhlet extraction, mechanical pressing, ultrasound‐assisted extraction, and microwave‐assisted extraction, are used to extract termite oil. Mechanical pressing yielded the significantly highest oil quantity (37.42%–38.50%) compared to Soxhlet and green extraction techniques (microwave and ultrasound‐assisted extraction methods). This is congruent with the emphasis of Tzompa‐Sosa et al. (2019), who found that the use of green extraction techniques usually yields a low oil quantity.

Microwave‐assisted extraction method yielded significantly higher oil quantity (24.91%–29.27%) than ultrasound and Soxhlet extraction methods (Table 1). According to Hao et al. (2021), microwave power induces localized heating in insect samples and serves as a driving factor for the destruction of the biomass matrix, enabling lipids to easily disperse out of the matrix and ultimately dissolve in the solvent. However, Hao et al. (2021) findings in black soldier fly larvae differed considerably from present findings; conventional Soxhlet extraction and microwave‐assisted extraction produced closely similar lipid quantities of 34.24% and 30.53%, respectively.

Ultrasound‐assisted extraction yielded a higher quantity of termite oil (20.83%–22.66%) than Soxhlet extraction (8.05%–18.92%). This result is congruent with the previous findings of Gharibzahedi and Altintas (2022) in lesser mealworm ( *Alphitobius diaperinus* L.) larvae, where ultrasound‐assisted extraction using ethanol/isopropanol yielded a significantly higher oil quantity (89.41%) than the method of conventional Soxhlet extraction with n‐hexane (60.09%). The aforementioned authors highlighted that the ethanol/isopropanol mixture easily penetrates the sample matrix to extract lipids and that the disruption, diffusion, and leaching out of lipids in ultrasound‐assisted extraction requires less energy than the conventional Soxhlet extraction method. Overall, the significantly lowest oil yield was observed in the Soxhlet/conventional extraction method (8.05%–18.92%). Relatedly, Laroche et al. (2019)'s study provided evidence that Soxhlet oil/crude fat extraction with hexane yielded the lowest fat quantity in house crickets, that is, 14.6%. In the present study, Soxhlet extraction yielded an oil content of 18.60% in *M. subhyalinus*. This yield was lower than the 47.03% oil produced through Soxhlet extraction by Ajayi (2012) from *M. subhyalinus* sourced from Nigeria.

### 4.2 Characterization of the Extracted Oil

#### 4.2.1 Color Characteristics

Color characteristics are a crucial component of oil quality, which significantly influences the sensory perception and consumer acceptability of edible insects and their products (Vanqa 2022; Vanqa et al. 2022) and is usually the first parameter considered when assessing food quality, even for edible oils (Tzompa‐Sosa et al. 2019). According to the L* a* b* C* H* scale (Table 2) and Figures 1 and 2, the extracted termite oils appeared clear, golden‐to‐light yellow liquids at room temperature (26°C ± 2°C), and no species differences were significant in the yellowness index of the oil. The present results align with the following: (i) Kinyuru's (2020) description of *M. subhylanus* visual oil appearance, (ii) Ajayi's (2012) description of *M. subhyalinus* oil; and (iii) Ekpo and Onigbinde's (2007) findings in *M. bellicosus*. According to Tzompa‐Sosa et al. (2019), the yellow color of insect oils is due to lipid‐soluble carotenoids, which could be de novo synthesized or sourced from the diet. In the present study, all extracted termite oils produced a very low redness index, with no significant differences among the termite species and oil extraction techniques. This result is similar to earlier observations by Tzompa‐Sosa et al. (2019) in four edible insect oils commercially reared in Europe (house cricket, lesser mealworm, yellow mealworm, and Dubia cockroach). The clear, golden‐to‐light yellow color of termite oils in the present study did not vary considerably from the typical appearance of most vegetable edible oils, and this attribute can enhance consumer acceptance if these oils are used as table oils and food ingredients (Tzompa‐Sosa et al. 2019).

#### 4.2.2 Solidification Temperature

Present findings indicate that the extracted termite oil had a solidification temperature of 10.1°C–12.9°C and no species differences were observed. This value is consistent with the oil solidification temperature of 10°C–14°C previously reported for *M. bellicosus* from Nigeria (Ekpo and Onigbinde 2007) and 8°C–12°C previously reported for *M. subhylanus* from western Kenya (Kinyuru 2020).

#### 4.2.3 Refractive Index

Insect oils have been reported to possess low refractive indices (Rahman et al. 2024). The refractive index of the extracted termite oil varied from 1.23 to 1.46, and significant differences were not evident between the extraction techniques and termite species. The present result is consistent with Ajayi (2012)'s refractive index of 1.465 in winged *M. subhyalinus* and Kinyuru (2020)'s refractive index of 1.35 in *M. subhyalinus*. However, the refractive index of *M. bellicosus* in the present study, which ranged from 1.35 to 1.43, was slightly higher than the 1.20 value reported by Ekpo and Onigbinde (2007) for *M. bellicosus* from Nigeria. Differences in the analytical techniques used in different studies could be responsible for the slightly higher values in the present study. According to Ekpo and Onigbinde (2007), the low refractive index of termite oil implies that this oil is less viscous at low temperatures and may thus be used in the pharmaceutical industry. Similarly, Ekpo et al. (2009) compared the physical and chemical characteristics of common oils, including linseed oil, olive oil, and arachis oil, with those of insect oils such as *M. bellicosus*; the specific gravity and refractive index of insect oil were lower, which implies that insect oils are lighter and have high oxidative stability.

#### 4.2.4 Iodine Value

Iodine value is considered a good index for measuring the degree of unsaturation of oils in a food sample (Ekpo and Onigbinde 2007; Mabossy‐Mobouna et al. 2020). The present study indicated a high iodine value of 80–121 g iodine/100 g oil. This result is comparable to the iodine value of (i) 83.51 g iodine/100 g oil in *M. subhyalinus* oil reported by Kinyuru (2020), (ii) 118.60 mg/g in *M. subhyalinus* oil reported by Ajayi (2012), (iii) 108 ± 0.15, previously reported in *M. bellicosus* (Ekpo and Onigbinde 2007), and (iv) 88.1, reported by Badanaro et al. (2018) for *M. bellicosus*. According to Kinyuru (2020) and Ekpo and Onigbinde (2007), a high iodine value indicates unsaturated oil. This was indeed proved true for the termite species in the present study, from our previous work (Khadijah et al. 2025); flour from the same termite species had more types of unsaturated fatty acids than saturated ones. Given this high iodine value and degree of unsaturation, we are convinced that the extracted termite oil is linked to a decreased risk of coronary heart disease, and thus a healthy oil for the human diet.

#### 4.2.5 Peroxide Value

Peroxide value of termite oil was low and ranged from 0.17 to 0.79 mg KOH/g. Generally, Soxhlet‐extracted oil produced the highest peroxide value, and no significant species differences were observed. The present results are in agreement with the previously reported peroxide value of 0.78 mg KOH/g in Ajayi (2012)'s *M. subhyalinus* oil. According to Kinyuru (2020), such a low peroxide value of insect oils compared to virgin oils such as palm oil and olive oil with higher peroxide values indicates that insect oils are less susceptible to rancidity and are more stable during food processing operations. The high concentration of antioxidants, especially α‐tocopherol, in insects has been highlighted as being responsible for low peroxide values (Kinyuru 2020).

#### 4.2.6 Saponification Value

From the present study, edible winged termite oil produced a saponification value of 101.81–113.92 mg KOH/g, and oil from *M. subhyalinus* had a significantly higher saponification value than oil from other termite species. Present findings are supported by the earlier results of Ajayi (2012), where the saponification value for *M. subhyalinus* oil was 108.45 mg KOH/g. On the contrary, the present result is very low compared to the saponification value of 193.40 ± 0.31, previously reported for *M. bellicosus* (Ekpo and Onigbinde 2007). Differences in analytical techniques used in each of these studies could be responsible for the wide variation observed in saponification values from different studies.

#### 4.2.7 Total Cholesterol Content

Sterols, particularly cholesterol, are essential for normal growth, reproduction, and metamorphosis in insects (Ekpo and Onigbinde 2007). The total cholesterol content of termite oils ranged from 24.98 to 39.00 mg/100 mL, and *M. bellicosus* oil produced the significantly highest total cholesterol content (up to 39.00 mg/100 mL). This result is comparable to (i) Kinyuru (2020)'s earlier reported total cholesterol content of 38.77 mg/100 mL in *M. subhyalinus* oil and (ii) Ekpo and Onigbinde (2007)'s earlier reported total cholesterol content of 47.18 mg/100 g lipid in *M. bellicosus* oil. However, differences in insect species and diet are responsible for the variations in sterol content in some insects (Ekpo and Onigbinde 2007). The total cholesterol content in the present study was low compared to the values reported for some conventional foods, notably those of animal origin, such as beef (50 mg/100 g), mutton (66 mg/100 g), lamb (66 mg/100 g), and veal (51 mg/100 g) (Kinyuru 2020; Williams 2007). The fact that low cholesterol content in food reduces the risk of coronary artery disease is well documented (Kinyuru 2020; Mabossy‐Mobouna et al. 2020), which makes termite oil a healthy edible oil. We were unable to benchmark cholesterol content values of the present study against the relevant food standards such as codex standard for named vegetable oils (CODEX STAN210‐1999), which gives the maximum permissible cholesterol levels in specific vegetable oils. This is mainly because the present termites were wild‐harvested, and as such, reported findings are likely to change for similar edible winged termite species that are reared on artificial/specific diets and under controlled environmental conditions. To date, a completely artificial termite rearing system has not yet been reported.

The extracted termite oils in the present study existed as fluids at room temperature (26°C ± 2°C), golden‐to‐light yellow odorless liquids, with low solidification temperature, refractive index, and total cholesterol content, which makes termite oil comparable to or even superior to many virgin oils in quality (Alen et al. 2017; Khadijah et al. 2024; Kinyuru 2009, 2020). Similar to virgin oils, the present study provides evidence that termite oils can also be extracted mechanically by pressing, using minimal heat, without the need for chemical treatment. Moreover, our previous work indicates that the present termite species have more types of unsaturated fatty acids than saturated ones (Khadijah et al. 2025), a characteristic common to virgin oils. Termite oil is thus an excellent candidate for industrial food processing, especially if artificial or semi‐natural rearing systems are developed to guarantee continuous supply and quality control from farm to fork (Khadijah et al. 2024). As liquids at room temperature, insect oils can find many uses as food‐grade lubricants and frying oils (Ekpo and Onigbinde 2007; Khadijah 2024; Kinyuru 2009, 2020). Therefore, the present study shows that termite oil has qualities that are preferred for table oils and oils used as food ingredients. Relatedly, Smetana et al. (2020) investigated the potential of insect‐based margarine production so as to find a replacement for the *trans*‐fat containing plant‐based margarine, which has been linked to health hazards; their findings found that insect lipids have the potential to replace up to 75% of plant lipids without compromising the spreading abilities and with better product coloring.

### 4.3 Chitosan Extraction From Different Termite Species Using Different Techniques

According to Ssekatawa et al. (2021), the demineralization and deproteinization degrees dictate chitosan's purity, which consequently influences chitosan's biological activities; for instance, adequately demineralized chitosan with an ash level of less than 1% has better biological properties.

#### 4.3.1 Demineralization Percentage

Our findings indicate that the defatted termite flour chemical demineralization percentage ranged from 80.05% to 83.12%, and no significant differences were observed among termite species (Table 3). Present results are lower than the 95.85% and 91.1% chemical demineralization percentages observed by Espinosa‐Solís et al. (2024) and Psarianos et al. (2022), respectively, in house cricket ( *Acheta domesticus* ) flour. The ash content of a sample is representative of its mineral quantity. The 4.3% ash content of house cricket flour (Espinosa‐Solís et al. 2024) versus the 6.44%–7.94% total ash content of the present termites is a convincing explanation for the higher demineralization percentage of house cricket flour compared with the present termite species. Present findings were, however, higher than the 58% chemical demineralization percentage obtained by Khayrova et al. (2019) for the black soldier fly ( *H. illucens* ) with an ash/mineral content of 7%. Differences in the temperature–time combinations used for demineralization in each of the two studies could be responsible for the observed differences in findings.

From the present results, Citric acid treatment showed the lowest demineralization percentage (70.42%–76.83%). Present results are comparable to those of Psarianos et al. (2022) in the house cricket *A. domesticus* , where the lowest demineralization percentage (70.5%) was recorded with citric acid treatment.

*L. lactis* fermentation of defatted termite flour yielded a demineralization percentage of 93.88% to 96.06% from different species, whereas microwave treatment yielded a demineralization percentage of 80.73%–84.01%. These results are slightly lower than the previously reported demineralization percentages of 97.3% and 85.8% from *L. lactis* fermentation and microwave treatment, respectively, from Psarianos et al. (2022)'s *A. domesticus* . Differences in the compositional analysis, especially the total ash content of these two insects, could be responsible for the observed slight differences.

#### 4.3.2 Deproteinization Percentage

In the present study, the chemical method produced the significantly highest deproteinization percentage (40.77%–43.71%). Present results are closely similar to the 43.23% chemical deproteinization percentage previously observed by Espinosa‐Solís et al. (2024) in house cricket flours. This might be a result of the closely similar crude protein content of 56.29% in house cricket flour (Espinosa‐Solís et al. 2024) and the 50.74%–57.96% crude protein content of the present termite species. Our chemical deproteinization percentage was, however, slightly lower than the 46% value obtained by Khayrova et al. (2019) for the black soldier fly ( *H. illucens* ). The low deproteinization percentage of enzymatic treatments compared to chemical treatment observed in the present study was already postulated by Mohan et al. (2020); the amount of protein remaining after the enzymatic method of protein degradation is higher and takes a longer reaction duration than the chemical method.

No significant differences manifested in the deproteinization percentage between bromelain treatment (20.47%–26.60%) and papain enzyme treatment (22.87%–27.23%). This is comparable to the results of Psarianos et al. (2022) for the house cricket, where no significant differences were observed between the two proteolytic enzyme deproteinization processes. The low deproteinization percentages of bromelain and papain observed in the present study are supported by Khayrova et al. (2021), where enzymatic deproteinization did not effectively eliminate proteins.

#### 4.3.3 Chitosan Yield

In the present study, the chemical method produced the significantly highest chitosan yield (6.05%–7.00%) than the two enzymatic treatments assessed. This result is comparable to the previously reported 6.58% chemically produced chitosan yield from the black soldier fly ( *H. illucens* ) pupal shell waste (Lagat et al. 2021), and is similar to the 5.7% chemically extracted chitosan yield from grasshoppers (Luo et al. 2019). However, the present results are very low compared to the 81.9% chemically extracted chitosan yield observed by Psarianos et al. (2022) in the house cricket, the 53% chemically extracted chitosan yield obtained by Khayrova et al. (2019) from the black soldier fly, and the 28.2% chemically extracted chitosan yield observed by Luo et al. (2019) from cicada slough. In Psarianos et al. (2022), the deproteinization step was performed before demineralization, which removes the protein layer, thereby exposing chitin for faster deacetylation. Ultimately, a high chitosan yield was obtained (No et al. 2003).

In the current study, bleaching and purification of chitin were conducted before deacetylation, whereas Khayrova et al. (2019) employed deacetylation before purification, which could be responsible for the variability in the chitosan yield from both the experimented termites and Khayrova et al.'s (2019) black soldier fly. According to Espinosa‐Solís et al. (2024) and Rehman et al. (2023), the variability of chitin and chitosan yields from different insects and/or species across publications is mainly because the extraction techniques from insect sources, including the conventional chemical method, have not been standardized as various chemical reagents and reaction conditions are used, for example, temperature–time combinations and molar concentrations, especially during demineralization and deproteinization processes. The absence of standard protocols for specific insects, including termites, makes a comparison of chitosan yield from different insects or species cumbersome. Similarly, Psarianos et al. (2022) and Rehman et al. (2023) highlighted that the amount of chitin and chitosan produced by insects varies due to differences in species, developmental stage, conditions under which the insect is raised, and the age of the insect. Moreover, Mohan et al. (2020) and Psarianos et al. (2025) emphasized that chitosan yield varied from one insect species to another. To the best of our knowledge, the current study is the first to report chitin extraction and chitosan production from edible winged termites, thus limiting our capacity to contrast the current results with previously published scientific works on the subject.

### 4.4 Characterization of the Extracted Chitosan

#### 4.4.1 Molecular Weight

The molecular weight of chitosan influences many physicochemical properties and biological behaviors such as hydrophilicity, solubility, and antimicrobial ability (Guarnieri et al. 2022; Rehman et al. 2023; Ssekatawa et al. 2021). Chitosan with a high molecular weight is likely to have poor water solubility, which consequently reduces its application in the food industry (Mohan et al. 2020). Additionally, Mohan and co‐workers highlighted that differences in the molecular weights of chitin and chitosan from different organisms are dictated by the source and extraction method employed.

The present results (Table 4) indicate that in all the termite species, enzymatically extracted chitosan produced significantly higher molecular weight (334.6 × 10^3^–366.0 × 10^3^ g/Mole) than chemically extracted chitosan (198.2 × 10^3^–271.2 × 10^3^ g/Mole). This result contrasts with the findings of Psarianos et al. (2022) in the house cricket, where biologically extracted chitosan produced lower molecular weight (86.8 × 10^3^ g/Mole) than the chemically extracted chitosan (103.4 × 10^3^ g/Mole). According to the aforementioned authors, the lower the molecular weight of chitosan, the higher its antimicrobial, antioxidant, and antitumor abilities. Similarly, Guarnieri et al. (2022), Psarianos et al. (2025), and Ssekatawa et al. (2021) highlighted that bacterial cell walls can be successfully penetrated by low‐molecular weight chitosan because it has a greater penetrative capability than high molecular weight chitosan, and thus a higher antimicrobial ability of the former. This implies that chemically extracted termite chitosan with a lower molecular weight has a higher antimicrobial ability than enzymatically extracted termite chitosan with a higher molecular weight. Indeed, this was the case during our experiment for the antimicrobial ability of the produced termite chitosan (Table 5), and in most termite species, chemically extracted chitosan exhibited stronger antimicrobial activity than the two enzymatically extracted chitosans (bromelain and papain‐extracted chitosan).

It is important to note that higher molecular weight chitosan, such as the enzymatically extracted termite chitosan in the current study, has been reported to exhibit antibacterial activity if used at high concentrations (Guarnieri et al. 2022; Rehman et al. 2023; Ssekatawa et al. 2021).

#### 4.4.2 Water Binding Capacity and Fat Binding Capacity

Water binding capacity (WBC) is the tendency of water to bind with hydrophilic materials and hold as much water as possible per gram of sample material against gravity, whereas fat binding capacity (FBC) represents the quantity of oil absorbed per unit of weight (Mohan et al. 2020; Zielinska 2022). WBC and FBC are essential characteristics of food ingredients used in food processing and applications as they directly affect the moisture, texture, flavor, and taste of food products (Vanqa 2022; Vanqa et al. 2022; Zielinska 2022). In the present study, WBC of chemically‐extracted chitosan ranged from 565% to 572%, whereas the FBC ranged from 238% to 257%. The WBC values obtained from termite chitosan are however lower than the WBC values of chemically‐extracted chitosan from cicada slough (795%), silkworm chrysalis (635%), mealworm (643%), and grasshopper (594%) in Luo et al. (2019)'s study. Except for grasshopper, whose chitosan's FBC (275%) was slightly higher than the present chemically‐extracted FBC, the other three insect chitosans in Luo et al. (2019)'s study were considerably higher than the present results.

In all the termite species investigated, WBC and FBC were significantly higher in chemically extracted chitosan than in bromelain and papain‐extracted chitosan (Table 4). According to Vanqa et al. (2022), FBC is the ability of proteins in a sample to physically bind to fat through capillary action. Therefore, chemically extracted chitosan could be a high flavor retainer owing to its high FBC and is useful in several food industry applications, such as in the processing of bakery products and sausages (Vanqa et al. 2022). To the best of our knowledge, this is the first study to report WBC and FBC of edible winged termite chitosan.

### 4.5 Termite Chitosan's Antimicrobial Ability

Chitosan has been reported to have potent antibacterial and antifungal properties by several authors (Abdelaziz et al. 2024; El‐Fakharany et al. 2025; Psarianos et al. 2022; Rehman et al. 2023). Chitosan is known for its biocompatibility, biodegradability, and ability to produce coatings/membranes that inhibit microbial proliferation (Albalawi et al. 2023; El‐Fakharany et al. 2025), making it a useful product for extending the shelf life and safety of fresh foods, particularly fruits and vegetables (da Silva Simoes et al. 2025). All chitosan samples produced from edible winged termite flours induced the formation of measurable inhibition zones against both bacteria (gram‐positive and gram‐negative) and yeast (Table 5). This finding is comparable to the result of Guarnieri et al. (2022), in which all chitosan samples produced from the black soldier fly induced the formation of measurable inhibition zones against *E. coli* and *Micrococcus flavus* at all tested concentrations. Relatedly, some termite extracts have been reported to offer antibacterial properties; for example, Afolayan et al. (2019)'s in vitro study which assessed the anti‐Salmonella ability from *M. bellicosus* extracts in n‐hexane and ethyl acetate revealed that *Salmonella typhi, Salmonella paratyphi* A, B, plus C were all inhibited by the African soldier termite. Furthermore, a natural antibacterial product was discovered in *Nasutitermes corniger* and commonly used in Brazilian traditional medicine (Coutinho et al. 2010).

In this study, varying degrees of antimicrobial activity were observed against *S. aureus* , *E. coli* , and *C. albicans* . The present results are similar to those of Lagat et al. (2021), in which chitosan from black soldier fly ( *H. illucens* ) pupal shell waste exhibited varying degrees of antibacterial action against *C. albicans* , *E. coli* , *Pseudomonas aeruginosa* , *S. aureus* , and *Bacillus subtilis* . Our findings further indicated that chitosan from most termite species produced the significantly highest antimicrobial activity against *E. coli* (gram‐negative bacteria), followed by *S. aureus* (gram‐positive bacteria), whereas the significantly lowest antimicrobial activity was observed in *C. albicans* (yeast). The present results of higher *E. coli* inhibition than *S. aureus* inhibition are supported by the previous results of Lagat et al. (2021), where black soldier fly chitosan was found to work better against gram‐negative bacteria than gram‐positive bacteria. According to Mohan et al. (2020), the underlying mechanism for this difference could be the breakdown of peptidoglycan brought about by interactions between the negatively charged microbial cell membranes and the positively charged chitosan molecules, which results in cell membrane collapse, intracellular component leakage, and ultimately cell death. In a related in vitro experiment, chitosan nanoparticles (CsNPs) and star anise extract loaded CsNPs produced detectable/measurable antibacterial activity against *S. aureus* associated with lung infection, that is, *S. aureus* inhibition zones of 6.80 mm and 15.41 mm were produced by CsNPs and star anise extract loaded CsNPs, respectively (Abdelaziz et al. 2024). Similarly, El‐Fakharany et al. (2025)'s study showed that a novel nanoplatform made of chitosan plus tungstate oxide with a surface functionalized by bovine‐milk lactoperoxidase enzyme had detectable activity against *S. aureus* , with an inhibition diameter of ~20.1 ± 1.2 mm. Present findings of the significantly lowest antimicrobial activity against *C. albicans* contrast with the findings of Lagat et al. (2021) in black soldier fly pupal exuviae. The current study is the first report on the antimicrobial activity of termite chitosan, which makes a comparison of current findings with the existing literature cumbersome.

Results of the present study indicate that in most termite species, chemically extracted chitosan exhibited significantly higher antimicrobial activity than the two enzymatically extracted chitosans (bromelain and papain‐extracted chitosan). This was because of the low molecular weight of chemically extracted chitosan (198.2 × 10^3^–271.2 × 10^3^ g/Mole) compared to the molecular weight of the enzymatically extracted chitosan (334.6 × 10^3^–366.0 × 10^3^ g/Mole). In this regard, Psarianos et al. (2022, 2025) and Ssekatawa et al. (2021) highlighted that the lower the molecular weight of chitosan, the higher its antimicrobial activity. Some previous studies have implicated the high ash content of chitosan (above 1%) as being responsible for chitosan's low antibacterial activity (Ssekatawa et al. 2021).

Except for *E. coli* where significantly higher and slightly considerable antimicrobial activity was observed, application of 1% acetic acid without chitosan produced very small that is, almost negligible microbial inhibition zones in the other two tested microbes ( *S. aureus* and *C. albicans* ) (Table 5). Our observations are similar to those of Guarnieri et al. (2022) during antimicrobial assessment of black soldier fly chitosan against *E. coli* and *M. flavus* ; 1% acetic acid used as a control showed slight inhibitory activity characterized by formation of undefined and unmeasurable inhibition zones. These observations suggest that 1% acetic acid has a weak microbial inhibitory effect and further confirm that the large‐diameter inhibition zones observed in the present study were largely contributed by termite chitosan not acetic acid. Although Lagat et al. (2021)'s study used 1% acetic acid alone (with no chitosan) as a positive control, their reported findings showed that in each tested microbe ( *S. aureus* , *P. aeruginosa* , *E. coli* , *B. subtilis* , and *C. albicans* ), 1% acetic acid produced shorter microbial inhibition zones than all the concentrations of black soldier fly chitosan used (0.5, 1.0, 2.5, and 5.0 g/mL chitosan). This further supports our suggestion that 1% acetic acid has a weak inhibitory effect against microbes. Relatedly, Ssekatawa et al. (2021) used 1% acetic acid as a negative control in the antimicrobial susceptibility assay of chitosan from Ugandan edible mushrooms, Nile perch scales, and banana weevils; no inhibitory effect was observed in either the carbapenem‐resistant or sensitive bacteria ( *E. coli* and *Klebsiella pneumoniae* ).

In our study, commercial chitosan from crustacean shells (positive control) showed very high antimicrobial activity (inhibition zones of 22.92–24.31 mm diameter) against all tested microbes. This finding is similar to the previous observations of: (i) Ssekatawa et al. (2021), in which commercial shrimp shell chitosan exhibited potent antibacterial activity against carbapenem‐resistant and sensitive bacteria ( *E. coli* and *Klebsiella pneumoniae* ), and (ii) Guarnieri et al. (2022), where commercial chitosan from crustaceans induced the formation of measurable inhibition zones against *E. coli* and *M. flavus* in the assessment of the antimicrobial ability of chitosan derived from the black soldier fly.

It is important to note that the antimicrobial activity of commercial chitosan in the present study was almost comparable to that of chemically extracted chitosan samples against *E. coli* for most termite species (Table 5). This implies that chemically extracted termite chitosan can be used to replace commercial chitosan from crustacean shells when antimicrobial activity against *E. coli* is desired. As expected, distilled water did not show any microbial inhibition zones (it was used as the negative control). Chitosan from edible insects can thus be used as an antimicrobial coating or film in food industries for prolonged storage of perishable products such as meat, fresh fruits, and vegetables (Rehman et al. 2023). Moreover, some studies have provided evidence that insect chitosan is a non‐toxic biofilm for food coating, with good water resistance, transparency, and vapor barrier characteristics (Liceaga et al. 2021; Rehman et al. 2023).

### 4.6 Safety Concerns and Potential Allergenicity of Insect‐Based Oils and Chitosan

Despite the promising potential of insect oils, their industrial processing and integration into mainstream food systems is still challenged by low consumer acceptance (Tanga et al. 2025) mainly due to unfamiliar flavor notes and perceived off‐flavors (Chang et al. 2025). Oil deodorization, which is commonly applied during refining to significantly reduce off‐flavors, is unfortunately associated with the removal of valuable components such as tocopherols, plus thermal degradation of heat‐sensitive color pigments like carotenoids (Tzompa‐Sosa et al. 2021) responsible for the desirable golden‐yellow appearance of most insect oils (Khadijah et al. 2025). Regarding food safety, bodies of wild‐harvested edible insects are likely to accumulate environmental pollutants such as lipophilic dioxins, which are a potential risk in insect‐based oils and lipids (Schluter et al. 2017). Generally, there is a paucity of research on the safety aspects and allergic concerns associated with insect‐based oils. This gap in knowledge presents a barrier to the broader application of insect oil as a viable substitute for conventional oils in culinary applications and commercial use.

Chitosan has been reported as an adsorbent for heavy metals such as Mercury, Lead, Cadmium, and Chromium, and this is attributed to chitosan's free amino groups exposed during deacetylation (Bailey et al. 1999). The possibility of insect‐derived chitosan to also exhibiting this metal adsorbent behavior thus merits investigation, and insect mass rearing needs to be carried out under carefully controlled conditions (Schluter et al. 2017). It is not known whether chitin fractions from insects contain undesired, insect‐specific contaminants whose removal would require additional technological processing stages.

Regarding chitosan allergenicity concerns, Schluter et al. (2017) and Romero (2025) highlighted that insect chitin has immunomodulatory potential; for example, mealworm chitin can enhance the formation of allergen‐specific immunoglobulin E antibodies, which trigger immediate hypersensitivity reactions in shrimp‐allergic patients. To this end, Schluter and co‐workers concluded that food allergy from insect consumption might be significantly reduced by processing methods like fermentation. However, Romero (2025) emphasized that both the conventional and novel food processing techniques currently available do not completely eliminate allergenic effects. Our search indicated that the World Health Organization and International Union of Immunological Societies (WHO/IUIS) database (www.allergen.org, last accessed on August 26, 2025) has not reported any allergen from the presently investigated species of edible winged termites (*M. subhylanus*, *M. bellicosus, P. spriniger*, and *O. lateritius*). Similarly, published literature has not yet reported any allergic fraction from the presently investigated species of edible winged termites or cross‐reactivity plus anaphylactic reactions from their consumption. However, the WHO/IUIS database has reported (but not yet published) one termite allergen (named Copt f 7–Tropomyosin) from the subterranean termite *Coptotermes formosanus* , whose route of exposure is through air/inhalation.

## 5 Conclusion

From the current study, mechanical pressing yielded the highest oil quantity, followed by microwave‐assisted extraction. Generally, the extracted termite oils appeared as clear, golden‐to‐light yellow liquids. The extracted termite oils had a high iodine value and were highly unsaturated, whereas the low peroxide value indicated that this oil was not easily susceptible to rancidity. Green oil extraction methods such as mechanical pressing, microwave, and ultrasound‐assisted techniques produced better oil results and could thus replace conventional Soxhlet extraction. There was considerable growth inhibition of *S. aureus* and *E. coli* when exposed to termite chitosan solutions at a concentration of 50 μL. This chitosan can be used to replace commercial chitosan from crustacean shells, especially when antimicrobial activity against *E. coli* is desired. The present findings demonstrate that the antimicrobial effects of termite chitosan have potential for the development of active edible coatings or films for food packaging applications. Present results focused on wild‐harvested edible winged termites, and as such, reported findings are likely to change for similar edible winged termite species that are reared on artificial diets and under controlled environmental conditions. To date, a completely artificial termite rearing system has not yet been reported. The present termite samples were sourced from local outdoor markets and thus associated with foodborne microbes of public health concern, such as *Escherichia coli* , *Staphylococcus* species, and *Listeria monocytogenes* (unpublished data—Manuscript currently under journal peer review).

## 6 Limitations

The present study did not assess the degree of deacetylation during the characterization of the extracted edible winged termite chitosan, which is among the parameters known to influence the biological activity of chitosan. Solvent recovery yield for both microwave and ultrasound‐assisted extraction methods was also not assessed in the present investigation.

## 7 Recommendations

In addition to comparing the yield/quantity of recovered oil from different extraction techniques, we recommend an analysis of how different extraction variables (e.g., temperature, time, solvent to flour ratio) influence both the oil yield and extraction efficiency with different oil extraction techniques. For potential industrial applicability, shelf life or oxidative stability assessment of termite oil from the investigated species is important. Further studies are needed to standardize the chitosan production protocol from edible winged termites to facilitate yield comparison across species and with other insect‐derived chitosan. Additionally, further studies on the antimicrobial ability of termite chitosan need to assess the minimum inhibitory concentration (below 50 μL) that will be just enough to cause microbial suppression.

## Author Contributions

**Babirye Khadijah:** conceptualization (lead), formal analysis (lead), investigation (lead), writing – original draft (lead), writing – review and editing (equal). **Ammar Ahmad Khan:** methodology (equal), supervision (equal), validation (equal), visualization (equal), writing – review and editing (equal). **Shahrul Razid Sarbini:** methodology (equal), supervision (equal), validation (equal), visualization (equal), writing – review and editing (equal). **Aqsa Abid:** data curation (lead), writing – review and editing (equal).

## Ethics Statement

The authors have nothing to report.

## Conflicts of Interest

The authors declare no conflicts of interest.

## Data Availability

All the data related to this article is included within the write‐up.
